# Kinlessness in Later Life: A Scoping Review

**DOI:** 10.1093/geroni/igaf122.3301

**Published:** 2025-12-31

**Authors:** Jianan Li, Claire McDonald

**Affiliations:** University of California, Los Angeles, Los Angeles, California, United States; University of Maryland Baltimore County, Baltimore, Maryland, United States

## Abstract

Shifting adult family structures—driven by rising childlessness, declining marriage rates, and increased life expectancy—have led to a growing population of kinless older adults, defined as those without a partner and children. This group comprises approximately 7% of the aging population in the United States. Despite the growing evidence on this population, research on their multidimensional well-being remains fragmented across disciplines. This scoping review synthesizes 17 existing articles to examine how definitions of kinlessness vary and to assess the well-being of this population using the PRISMA-ScR guidelines. The different definitions highlight the limitations of framing kinlessness solely in terms of traditional family structures, simple measures of kin presence, and deficit-based perspectives. Empirical evidence highlights a complex pattern of vulnerabilities. Physically, kinless older adults report poorer self-rated health despite often having fewer comorbidities, with greater reliance on formal care services and more unmet needs. Mentally, their unpartnered status contributes to greater loneliness and poorer subjective mental health, though aging itself may mitigate these effects as individuals adapt. Socially, they have less tangible support but maintain distinct—not necessarily fewer—social connections compared to those with close kin. However, well-being varies significantly by age, gender, education, and cultural context. Future research should examine pathways to kinlessness through a life-course perspective, explore resilience mechanisms, and consider intersecting social determinants to better support this diverse population. A nuanced understanding of their experiences is essential for addressing their needs in aging societies.

